# Intestinal Fatty Acid Binding Protein (I-FABP) as a Prognostic Marker in Critically Ill COVID-19 Patients

**DOI:** 10.3390/pathogens11121526

**Published:** 2022-12-13

**Authors:** Maciej Tyszko, Małgorzata Lipińska-Gediga, Anna Lemańska-Perek, Katarzyna Kobylińska, Waldemar Gozdzik, Barbara Adamik

**Affiliations:** 1 Clinical Department of Anesthesiology and Intensive Therapy, Wroclaw Medical University, Borowska 213, 50-556 Wroclaw, Poland; 2 Department of Chemistry and Immunochemistry, Wroclaw Medical University, Marii Sklodowskiej-Curie 48/50, 50-369 Wroclaw, Poland; 3 Faculty of Mathematics, Informatics and Mechanics, University of Warsaw, Banacha 2, 02-097 Warsaw, Poland

**Keywords:** COVID-19, I-FABP, citrulline, biomarker, intestinal injury, sepsis, intensive care

## Abstract

Gastrointestinal symptoms are common in critically ill COVID-19 patients. There is currently no generally recognized method of assessing gastrointestinal injury in unconscious or sedated intensive care unit (ICU) patients. I-FABP (intestinal fatty acid binding protein) and citrulline have previously been studied as potential biomarkers of enterocyte damage in various gastrointestinal tract diseases, and changes in the levels of these markers may reflect intestinal wall damage in COVID-19. Patients with critical COVID-19, with diagnosed sepsis, or septic shock requiring ICU treatment were included in the study. Blood samples for citrulline and I-FABP were taken daily from day 1 to 5. I-FABP levels were significantly higher in patients who eventually died from COVID-19 than in survivors, and the optimal I-FABP cut-off point for predicting 28-day mortality was 668.57 pg/mL (sensitivity 0.739, specificity 0.765). Plasma levels of I-FABP, but not citrulline, were associated with significantly higher mortality and appeared to be a predictor of poor outcome in multivariate logistic regression analysis. In conclusion, I-FABP seems to be an effective prognostic marker in critically ill COVID-19 patients. Assessing mortality risk based on intestinal markers may be helpful in making clinical decisions regarding the management of intestinal injury, imaging diagnostics, and potential surgical interventions.

## 1. Introduction

The severe acute respiratory syndrome caused by the severe acute respiratory coronavirus 2 (SARS-CoV-2) virus was a serious challenge for healthcare systems during the COVID-19 pandemic, especially for already overcrowded intensive care units (ICU). In some patients hospitalized due to a COVID-19 infection, rapid deterioration of the respiratory system function and the development of sepsis were observed, both conditions requiring immediate ICU treatment [1,2]. The current definition defines sepsis as life-threatening organ dysfunction caused by a dysregulated host response to infection [2]. Sepsis can be the final pathway of infection caused by a variety of pathogens, including bacteria, fungi, viruses, and parasites. According to the World Health Organization, viral pathogens such as seasonal influenza viruses, dengue viruses, and highly contagious pathogens of public health concern such as avian and swine flu viruses, Middle East respiratory syndrome coronavirus, Ebola, yellow fever viruses, and, most recently, severe severe acute respiratory coronavirus 2 can lead to the development of sepsis [3]. Prior to the COVID-19 pandemic, viral sepsis was rare among ICU patients with occurrence rates ranging from 1.0% to 6.3% in different regions [4,5,6]. Unfortunately, sepsis of viral origin has become a daily survival struggle in the ICU, as COVID-19 can develop from a mild infection to septic shock within hours. The results of a recent meta-analysis showed that the prevalence of sepsis associated with the coronavirus disease in the ICU was 77.9% [7]. The lungs are the main target for the SARS-CoV-2 virus, and respiratory failure remains the most important problem on ICU admission; however, later some patients develop failure of other vital organs leading to the development of multiple organ failure and often death [8,9,10,11].

Activation of the systemic inflammatory reaction caused by severe lung infection can lead to a disruption of the integrity of the intestinal barrier (the gut–lung axis,) increasing its permeability to intestinal microorganisms and endotoxins. It was shown by Dickson et al., some intestinal bacteria (e.g., *E. faecalis*) can be isolated from post-sepsis lungs, which seems to prove the role of intestinal translocation in the development of ARDS and sepsis [12,13]. Gastrointestinal symptoms may occur with respiratory symptoms of COVID-19 [14]. In a study by Kang et al. nearly half of COVID-19 patients were shown to have severe gastrointestinal problems in addition to respiratory [15]. In a population of COVID-19 patients treated at the ICU, a common problem was dysfunction of the gastrointestinal tract and feeding intolerance (56%) manifested by large gastric residual volume, abdominal distension, and vomiting [16]. In addition, gastrointestinal problems in these patients were associated with poor treatment outcomes, including development of cardiac, renal, hepatic, and hematologic complications, longer hospital stay, and an increased risk of death. The mechanisms contributing to the deterioration of the intestinal barrier in the course of COVID-19 are not fully understood and have been the subject of many studies. The SARS-CoV-2 virus can directly infect the epithelial cells of the intestinal tract, causing changes in tissue structure and breakdown of the epithelial barrier [17] It was previously confirmed that the metallopeptidase, angiotensin-converting enzyme 2 (ACE2) is a cell receptor for SARS-CoV viruses including SARS-CoV-2 [18,19]. The ACE 2 receptor shows a high level of expression in the gastrointestinal system and it has been shown that receptor ACE2 and the SARS-CoV-2 viral protein nucleocapsid stained positive mainly in the cytoplasm of gastrointestinal epithelial [20,21]. 

That is why it is so important to monitor the function of the gastrointestinal tract in ICU patients. Monitoring methods are limited and there is currently no generally recognized method of assessing gastrointestinal injury in unconscious or sedated ICU patients [22]. Clinical evaluation (e.g., an assessment of the presence of abdominal pain, distention, bowel sounds, diarrhea, bleeding) is commonly carried out but does not provide complete information on intestinal function. There is currently no validated score to assess gastrointestinal dysfunction in ICU patients, but two scores, the AGI (Acute Gastrointestinal Injury) score and the GIDS (Gastrointestinal Dysfunction) score, have been developed and await validation [23,24,25].The AGI score, first introduced a few years ago, aims to assess gastrointestinal malfunction in ICU patients based on gastrointestinal symptoms and their severity. However, it is a subjective descriptive assessment, and it may not be clear to the attending physician in the ICU how to grade an individual patient; for example, a patient with a rapidly deteriorating clinical condition may have a higher AGI score, despite having the gastrointestinal symptoms of a less severely ill patient. Therefore, a modified version of the AGI score, the GIDS score, has been recently proposed [24]. The clinical utility of this tool must be validated prospectively in future studies before it can be recommended for clinical use in ICU patients [23,24]. 

In addition to clinical evaluation, numerous endogenous proteins have been proposed as biomarkers of intestinal injury, but so far none have been routinely used in ICU patients. In this study, citrulline and I-FABP (intestinal fatty acid binding protein) were of interest as potential prognostic factors for treatment outcomes in ICU patients diagnosed with a COVID-19 infection. I-FABP is a cytosolic protein which plays a key role in the cellular uptake and metabolism of fatty acids in enterocytes and is released from cells in cases of injury [26]. Under physiological conditions, only small amounts of I-FABP are found in the bloodstream (57–310 pg/mL); therefore, an elevated level of circulating I-FABP may reflect intestinal wall damage [27]. The results of a previously published meta-analysis indicate that serum I-FABP measurements may be useful in the diagnosis of acute intestinal ischemia, with an AUC of 0.86, a sensitivity of 0.80, and a specificity of 0.85 [28]. I-FABP measurements can be especially useful in emergency situations as a quick and less invasive assessment; however, biomarker results should always be evaluated in parallel with histopathological and clinical findings. The diagnostic and prognostic potential of early necrotizing enterocolitis (NEC) markers, including markers of intestinal epithelial damage (intestinal fatty acid binding protein and liver fatty acid binding protein) and markers of excessive inflammatory response such as serum amyloid A, has been confirmed in neonates with suspected NEC [29]. Both epithelial damage and inflammation markers were significantly higher in those infants who later developed NEC, as early as 6 h after suspected NEC, suggesting that intestinal mucosal damage and a strong inflammatory response are detectable even before the onset of NEC symptoms. In the onset of heart failure, low cardiac output, splanchnic circulation congestion, and reduced intestinal blood perfusion can lead to disruption of the mucosal barrier and increased intestinal permeability [30]. In patients with acute heart failure, elevated I-FABP was associated with significantly worse clinical outcomes, including increased incidence of death, left ventricular assist device placement, or heart transplantation [31]. In another study, concomitant increases in serum I-FABP and endotoxin levels were observed in patients undergoing cardiac surgery with cardiopulmonary bypass, implying ischemia-reperfusion injury of the intestinal mucosa; the magnitude of the changes in the intestines depended on the duration of the cardiac bypass time [32].

The second protein of interest to us as a potential biomarker of intestinal damage was citrulline. It is an amino acid produced almost entirely by enterocytes from glutamine; therefore, its concentration may reflect the function and mass of the epithelial cells in the lining of the small intestine [33]. The importance of citrulline as a marker of intestinal wall damage was previously demonstrated in animal models, including models of sepsis, viral enteritis, and intestinal damage from chemotherapy [34,35,36]. In a rat model of sepsis, blood levels of citrulline were significantly reduced, with clear damage to the intestinal mucosa confirmed by histology [36]. 

Only few studies have focused on the usefulness of measuring intestinal injury biomarkers in intensive care patients, and the role of I-FABP and citrulline as potential biomarkers of intestinal cell damage in the most severe COVID-19 infections remains to be established [37,38,39,40]. 

In this study, we analyzed changes in the level of biomarkers of gastrointestinal damage in COVID-19 patients with the most severe symptoms who required immediate ICU treatment. We aimed to define a model utilizing the new biomarkers along with routinely monitored clinical and laboratory parameters to predict the risk of mortality in the analyzed cohort. Being able to detect an intestinal injury early can facilitate diagnosis, treatment, and decision making, particularly in deciding to initiate enteral nutrition in ICU patients.

## 2. Materials and Methods

### 2.1. Patients

We conducted a single-center prospective observational study on consecutive adult patients with SARS-CoV 2-induced sepsis or septic shock admitted to the ICU of the University Hospital between January 2020 and January 2021. The inclusion criteria were as follows: (1) a SARS-CoV2 infection confirmed by a positive result of real-time reverse-transcriptase polymerase chain reaction assay of nasal or pharyngeal swab probes prior to ICU admission, (2) critical COVID-19 according to WHO interim guidance [41], and (3) a diagnosis of sepsis or septic shock as defined by the SEPSIS-3 definition on admission to the ICU [2]. The exclusion criteria were as follows: (1) age < 18 years old and (2) a history of previous abdominal surgery or previous severe gastrointestinal disabilities (chronic inflammatory diseases, such as *ulcerative colitis,* Lesniowski-Crohn disease, viral hepatitis). Severe gastrointestinal inflammatory diseases may alter the levels of intestinal biomarkers [42,43]. The study protocol was accepted by the local Bioethical Committee (No. KB—822/2018). Written informed consent was obtained from the patient or a legally authorized representative. The study protocol complies with the 1975 Declaration of Helsinki as revised in 1983 [44].

All patients were treated according to the standard ICU procedures as described in the Surviving Sepsis Campaign guidelines [22,45]. Patients were transferred to the ICU from the emergency room or another hospital ward immediately after intubation. ARDS from SARS-CoV 2 infection was treated according to standard practice and an ECMO evaluation was made in the event of a rapid deterioration in respiratory function unresponsive to treatment. Empirical antibiotics were administered immediately after ICU admission and blood cultures and other relevant samples were taken regularly to detect any co-infections. Renal replacement therapy was implemented as needed.

### 2.2. Data Collection

Clinical data from each patient were collected daily from day 1 to 5. The clinical status of the patients was determined with the Acute Physiology and Chronic Health Evaluation (APACHE) II score on admission to the ICU and the Sequential Organ Failure Assessment (SOFA) score was used for daily monitoring of organ function. Both scores are standard prediction tools used at the ICU for septic patients. The APACHE II scale is based on the values of clinical and laboratory parameters (inhaled oxygen fraction, oxygen partial pressure, body temperature, mean arterial pressure, blood pH, heart rate, respiratory rate, serum sodium, serum potassium, serum creatinine, hematocrit, white blood cells, and Glasgow score), disease-related variables (history of severe organ failure or immunodeficiency), and it takes into account the type of ICU admission (elective/emergency). The SOFA scale enables the monitoring of the patient’s clinical condition on the basis of indicators of the functioning of the following systems: respiratory (PaO_2_/FiO_2_ ratio), cardiovascular (mean arterial pressure and the dose of vasopressors), hepatic (bilirubin concentration in the blood), coagulation (platelet count), renal (creatinine concentration in the blood/urine output), and neurological (Glasgow coma scale). The prevalence of organ dysfunction in patients with COVID-19 on admission to the ICU was calculated based on the SOFA scores. Additionally, the gastrointestinal injury (AGI) score and feeding intolerance described as high gastric residual volumes (GRV > 500 mL) were evaluated daily. All laboratory parameters were measured at the certified hospital laboratory. Mortality at 28 days was recorded. Data were recorded on the length of stay in the ICU and in the hospital.

### 2.3. Biomarker Analysis

Blood samples for determining plasma citrulline and I-FABP (intestinal fatty acid binding protein) were collected (2.7 mL, tubes containing 0.109 M sodium citrate as an anticoagulant, BD Vacutainer, BD, NJ) via a routinely inserted arterial cannula on ICU admission and on days 2, 3, 4, and 5. Plasma was separated immediately after centrifugation at 2000× *g* for 10 min and stored at –70 °C. I-FABP protein was analyzed using a commercially available ELISA kit (Quantikine ELISA Human FABP2/I-FABP Immunoassay, R&D Systems, Minneapolis, MN, USA). Citrulline was analyzed using a commercial ELISA kit (Human citrulline ELISA Kit, Cusabio Biotech Co., LTD, Houston, TX, USA). 

### 2.4. Statistical Analysis

We used Statistica 13 software (StatSoft, Inc., Tulsa, OK, USA) and the R 3.6.01: R Core Team (2013) to perform the analysis. Continuous variables are reported as mean values ± standard error and minimum–maximum and categorical as frequencies with percentages. The distribution was not normal based on the Shapiro–Wilk test and the analysis was performed with nonparametric tests. The Mann–Whitney U test was used to compare continuous variables between study groups at each time point. Categorical variables were analyzed using the Chi-square test or Fisher exact test. A comparison of the predictive accuracy of the biomarkers measured on admission to the ICU was made using receiver operating characteristic curve (ROC) analysis, by calculating the area under the curve (AUC), including 95% confidence intervals (CI), to determine sensitivity and specificity. Youden’s statistic was used to select the optimum cut-off point. Survival analysis of time to death was performed using the Kaplan–Meier curve and a Chi-square test. Univariate and multivariate logistic regression analysis was performed to evaluate the association between baseline values of the studied biomarkers of gastrointestinal damage, other parameters and covariates, and ICU outcome. Potential 28-day mortality prognostic variables were first selected based on their ease of measurement on ICU admission or their previously demonstrated role as a predictor of mortality. The results were reported as an odds ratio (OD) and 95% confidence intervals (CI). The first prepared model included biomarkers and all selected variables. The collinearity of the variables was tested and collinear features were excluded from the analysis. The choice of the best model was proposed based on the Akaike information criterion and the backward selection of the model. All the tests were conducted with a 5% significance level.

## 3. Results

### 3.1. Study Population

During the 12-month study period, 69 consecutive COVID-19 patients admitted to the intensive care unit were screened for inclusion criteria; of these, 42 met the criteria and 27 were excluded due to prior abdominal surgery or chronic intestinal disease. Out of 42 patients, in 2 the level of I-FABP and citrulline was not measurable due to high hemolysis of the sample and ultimately 40 patients were included in the analysis. The study group consisted of 15 adult females and 25 adult males; the mean age of the patients was 60.12 ± 15.5 years (28–90). Most of the patients (72%) were admitted to the ICU directly from the Emergency Department, and 28% were transferred from another hospital ward. The SARS-CoV2 virus was detected in all patients prior to admission to the ICU, and the diagnosis was confirmed in all patients upon admission to the ICU. All patients had severe respiratory failure secondary to COVID-19; ARDS was diagnosed in 85% of patients and pneumonia in 15%. Septic shock was the second-most-common dysfunction (40%) diagnosed on ICU admission, renal dysfunction requiring renal replacement therapy in 25% of patients, liver dysfunction in 13%, coagulopathy in 15%, and CNS dysfunction in 25%. The APACHE II score used to characterize patient morbidity on admission to the ICU was 17.2 ± 1.0 pts. (6.0–33.0), and the SOFA score calculated to assess the clinical status of patients was 8.0 ± 0.4 pts (3.0–13.0). The mean length of ICU stay was 19 days (2–106) and the 28-day mortality rate was 57%. The baseline characteristics of the study group divided into Nonsurvivors and Survivors based on 28-day mortality are presented in Table 1.

At ICU admission, all patients in our study required immediate support of the respiratory system, including 29 patients (73%) who were treated with mechanical ventilation and 11 (27%) with mechanical ventilation and V-V ECMO. Ten patients (25%) had renal replacement therapy administered at ICU admission. According to the Sepsis-3 criteria, sepsis on admission to the ICU was diagnosed in all patients, and 40% of patients presented with septic shock. The results of laboratory tests on admission to the ICU are presented in Table 2. Higher creatinine and lactate levels were found in Nonsurvivors compared with Survivors and other parameters were similar in both groups. 

### 3.2. Acute Gastointestinal Injury Score and Feeding Intolerance

The Acute Gastrointestinal Injury (AGI) score was used to monitor gastrointestinal dysfunction in ICU patients. On ICU admission, the overall incidence of AGI grade I/II was 100%. On subsequent days, most patients (>90%) had an AGI grade I/II and less than 10% had AGI III/IV. The incidence of feeding intolerance (gastric residual volumes > 500 mL) was 8% on ICU admission and increased on subsequent days. Differences in the distribution of the AGI scores and feeding intolerance rates between Survivors and Nonsurvivors were not significant (*p* < 0.05). The distribution of AGI scores and the incidence of food intolerances assessed on days 1–5 are presented in Table 3. 

### 3.3. Differences in I-FABPand Citrulline Levels between Survivors and Nonsurvivors

**Figure 1:**
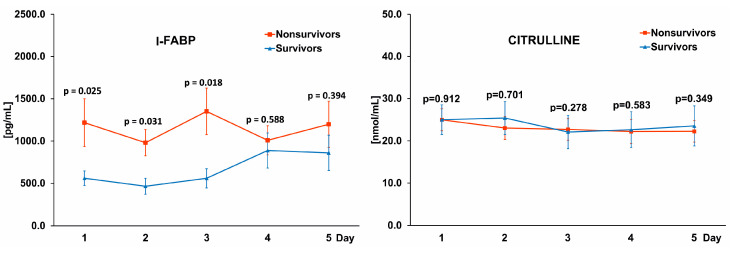
Graphs comparing the levels of I-FABP (intestinal fatty acid binding protein) (**left** panel) and citrulline (**right** panel) in the blood of Nonsurvivors and Survivors. The differences in the levels of biomarkers between study groups on corresponding days were marked with a *p*-value.

Blood samples taken every morning from the first to the fifth day of ICU treatment were used to analyze changes in I-FABP and citrulline levels in the course of the COVID-19 infection. I-FABP levels were higher in Nonsurvivors (1217.6 ± 282.1, 980.5 ± 155.3, 1350.8 ± 275.2, 1008.7 ± 172.2, 1198.1 ± 273 pg/mL, respectively on days 1–5) than in Survivors (560.0 ± 86.3, 465.2 ± 93.5, 550.1 ± 114.3, 88.7 ± 207.7, 860.0 ± 209.1 pg/mL, respectively on days 1–5), with statistically significant differences on days 1–3 and insignificant differences on days 4–5 (Figure 1, left panel). This pattern was not observed with citrulline, where similar levels were noted in Nonsurvivors (24.9 ± 2.6, 23.0 ± 2.79, 22.7 ± 2.5, 22.2 ± 2.8, 22.2 ± 2.5 nmol/mL, respectively on days 1–5), and Survivors (25.0 ± 3.5, 25.4 ± 3.9, 22.1 ± 3.9, 22.6 ± 4.3, 23.5 ± 4.7 nmol/mL, respectively on days 1–5) throughout the observation period (Figure 1, right panel). 

### 3.4. Prediction of Mortality

**Figure 2:**
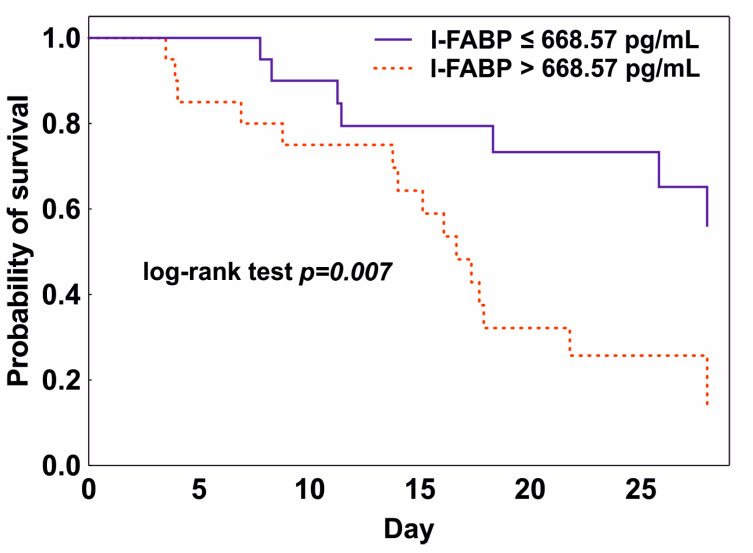
Kaplan–Meier curve showing 28-day survival stratified for patients with a baseline level of I-FABP ≤ 668.57 pg/mL (blue line) and >668.57 pg/mL (red line).

In the ROC curve analysis, the baseline I-FABP showed an ability to predict 28-day mortality with an AUC of 0.710 (95% CI 0.547–0.873, *p* = 0.011). The optimal threshold value for the baseline I-FABP was 668.57 pg/mL (sensitivity 0.739, 95% CI 0.376–0.740; specificity 0.765, 95% CI 0.274–0.863). The calculated threshold was used for the Kaplan–Meier analysis, which confirmed a significantly lower 28-day survival in the group with a baseline I-FABP above 668.57 pg/mL (log-rank test *p* = 0.007) (Figure 2). The ROC curve of the baseline citrulline concentration did not discriminate between Survivors and Nonsurvivors (*p* > 0.05).

In addition, a univariate and multivariate logistic regression analysis was performed to create a model predicting 28-day mortality in the analyzed cohort. The choice of the variables from the set of biomarkers measured on admission to the ICU (I-FABP, citrulline, WBC, procalcitonin, CRP, creatinine, and lactate), and covariates (age, sex, APACHEII and SOFA scores, shock on admission) was determined by the minimizing of the Akaike information criterion and the backward selection of the model. Collinear covariates were excluded from the analysis. Therefore, the only variables that were included in the final model were the initial APACHEII, I-FABP, PCT, creatinine and the presence of septic shock on admission to the ICU. An initial high level of I-FABP (>668.57 pg/mL), a high APACHEII score, and the presence of shock were significant predictors of bad outcome. The initial PCT and creatinine had no statistical significance in the model. The results of the univariate and multivariate logistic regression analysis are presented in Table 4.

### 3.5. Differences in I-FABP Levels between Patients with Sepsis and Shock

**Figure 3:**
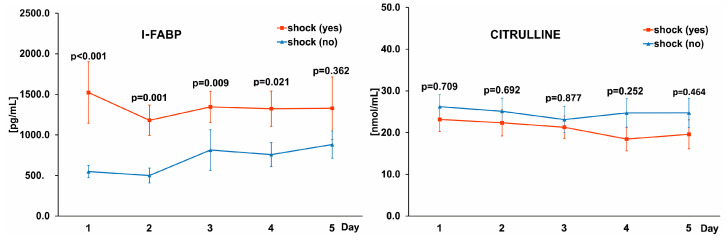
Graphs comparing the levels of I-FABP (**left** panel) and citrulline (**right** panel) in the blood of patients with and without septic shock. The differences in the levels of biomarkers between study groups on corresponding days were marked with a *p*-value.

Impaired blood flow in the gastrointestinal mucosa in septic shock can lead to damage to intestinal cells and the release of cytosolic proteins into the bloodstream. All the studied patients had sepsis on admission to the ICU and 40% were diagnosed with shock. Moreover, a diagnosis of shock was a significant predictor of mortality in a multivariate logistic regression analysis with an odds ratio of 89.58. We tested whether the concentrations of I-FABP and citrulline were different between COVID-19 patients with and without shock. Indeed, I-FABP levels were higher in the group of patients presenting with shock compared with the non-shock patients throughout the study period and citrulline levels were similar (Figure 3).

## 4. Discussion

Previously, it was shown that the SARS-CoV-2 virus can directly infect intestinal cells through the angiotensin-converting enzyme 2 to gain access to enterocytes [17]. As a consequence, some COVID-19 patients may have mild to severe symptoms of intestinal dysfunction. We focused on two issues: (1) monitoring intestinal cell damage with specific biomarkers I-FABP and citrulline, and (2) the relationship between the level of these biomarkers and mortality. The study included patients with critical COVID-19 with the most severe symptoms requiring ICU treatment. All patients required mechanical ventilation; ARDS was diagnosed in 85% of patients and pneumonia in 15% on admission to the ICU. Our results showed that damage to the intestinal cells is a potential factor that could contribute to the outcome of treatment in COVID-19 patients. Plasma levels of I-FABP, but not citrulline, were associated with significantly higher mortality and appeared to be a predictor of poor outcome in multivariate logistic regression analysis. I-FABP levels were significantly higher in patients who eventually died from COVID-19 than in survivors, and the optimal I-FABP cut-off point for predicting death was 668.57 pg/mL.

SARS-CoV-2 RNA mainly infects lung cells; however, fecal swabs testing positive for SARS-CoV-2 RNA occurred in 30–50% of patients [46]. The virus can cause intestinal inflammation and changes the permeability of the intestinal wall, making absorption by enterocytes difficult [47]. The results of gastrointestinal imaging in COVID-19 infections showed changes of varying severity—thickening of the intestinal wall, possible hyperemia and mesenteric thickening, and intestinal ischemia [48]. There are several methods for assessing intestinal permeability. Oral administration of molecules labeled with radioisotopes or indigestible sugars such as lactulose and mannitol have been used to determine intestinal absorption. In some studies, gastric tonometry has been used to measure gastric intramucosal pH and gastric partial pressure of carbon [49]. There is currently no standard procedure for assessing intestinal permeability in patients treated in the intensive care unit. Most of them are unconscious or sedated, and kidney failure is common; therefore, methods for determining the urinary excretion of orally administered tracer molecules cannot be used. 

Various endogenous proteins have been proposed as biomarkers of intestinal permeability, but it has not yet been established which indicators are most reliable in clinical practice. I-FABP and citrulline have previously been studied as potential biomarkers in various gastrointestinal tract diseases. In Crohn’s disease, serum levels of I-FABP in patients with active disease were significantly higher than in patients in remission and in the control group, indicating the potential utility of I-FABP as a marker of disease severity [50]. Elevated levels of I-FABP associated with villous atrophy were recorded in patients with untreated coeliac disease and a significant improvement of enterocyte function was seen in a majority of patients several months after the initiation of appropriate nutritional treatment [51]. The usefulness of I-FABP as an early biomarker in detecting intestinal damage has been assessed in patients with multiple trauma, where delayed diagnosis of intestinal injury might increase the risk of complications, including sepsis [27]. I-FABP levels were significantly higher in trauma patients with intestinal injury compared with the non-injured group (2101.0 pg/mL vs. 351.4 pg/mL, *p* < 0.05), indicating that I-FABP could be used as an early biomarker for the detection of intestinal injuries. Citrulline, another potential marker of the function of small intestinal epithelial cells, is produced almost entirely by enterocytes from glutamine. According to the previously published report, healthy subjects have plasma citrulline concentrations between 20 and 60 mmol/L, with a mean of 40 mmol/L [52]. The results of a meta-analysis indicate that low citrulline levels can be a diagnostic indicator of acute and chronic intestinal failure, reflecting a reduced enterocyte mass and, to a lesser extent, impaired intestinal absorption [53]. In a pediatric population, citrulline was established as an objective, easily measurable indicator for mucosal barrier injury in patients with chemotherapy induced mucosal barrier injury [54]. In our study, the mean citrulline concentration measured at ICU admission was lower than previously reported values in healthy subjects (25.0 nmol/mL vs. 40 nmol/mL, respectively), indicating enterocyte damage [52]. The lower citrulline values observed in patients with sepsis in our study are consistent with previous reports. Ware and colleagues showed that citrulline levels decreased in sepsis patients, especially those who developed acute respiratory distress syndrome [55]. In another study, similarly low levels of citrulline were reported in the general population of ICU patients; interestingly, using a threshold of 20 nmol/mL (which is the lower level of the normal range for plasma citrulline concentration), citrulline levels were not associated with 28-day mortality [38]. In our study, citrulline was not statistically significant in predicting 28-day mortality and was not included in the multivariate regression model. This can be explained by the fact that COVID-19 patients admitted to the ICU had lower levels of citrulline already on admission compared with the values measured in healthy people (40 nmol/mL); therefore, we were unable to observe a decrease in concentration during the 5-day ICU observation. In addition, a recent study of metabolic profiles in the serum of COVID-19 patients showed already significant changes in amino-acid metabolism at the time of admission to the hospital [56]. The authors found that l-glutamine, the main precursor of citrulline produced in the intestines, is the most altered amino-acid with reduced concentration in the acute phase of COVID-19. Thus, citrulline production may be limited by glutamine availability, potentially leading to lower baseline citrulline levels in critically ill COVID-19 patients. The beneficial effects of glutamine supplementation in COVID-19 have been evaluated in several studies where adding enteral L-glutamine to regular nutrition reduced hospital stay and improved outcomes [57].

The usefulness of markers of intestinal injury was also evaluated in viral infections. HIV infects and destroys the immune system of the gastrointestinal tract and causes structural abnormalities in the intestinal mucosa [58,59]. Changes in I-FABP levels as a biomarker of intestinal barrier dysfunction in acute and chronic HIV infections were investigated. Skowyra et al. measured I-FABP serum levels in HIV-infected patients and confirmed that HIV caused damage to the intestinal mucosa when compared with healthy volunteers (2100 vs. 1260 pg/mL) [60]. In another study it was found that high I-FABP was associated with worse immune function, increased inflammation, and viremia in chronically untreated HIV infections [61]. There are only a few published studies with findings on biomarkers of intestinal barrier dysfunction in COVID-19 patients, especially among ICU patients who are at the highest risk of death, and the results of these have been inconsistent. Saia et al. observed significantly increased levels of I-FABP indicating damage to enterocytes in SARS-CoV-2 infections [62]. In addition, critically ill and non-survivors showed higher levels of I-FABP compared with patients with a less severe clinical course. Giron et al. investigated markers of intestinal wall damage in COVID-19 patients using a multiplex assay [37]. They found that COVID-19 infection was associated with elevated levels of markers of tight junction permeability in the digestive tract, including zonulin and hREG3A (regenerating family member 3 alpha) protein, a marker of intestinal stress. In addition, the markers of disrupted intestinal barrier integrity correlated with markers of systemic inflammation. Giron et al. did not show significant increases in the levels of I-FABP, which may suggest that the changes in the intestinal wall were not associated with enterocyte death in the studied cohort. The lack of a significant increase in I-FABP could be explained by the clinical condition of the patients: most of the patients enrolled in Giron’s study had mild to moderate symptoms (outpatients, hospitalized in regular wards), and only a few patients were critically ill. In our study, all patients were treated in the ICU for the most severe symptoms of COVID-19; all had respiratory failure and required mechanical ventilation (ARDS diagnosed in 85%), nearly half had septic shock, and 30% required renal replacement therapy to treat acute kidney failure. In this cohort, the higher levels of I-FABP in COVID-19 patients were associated with a diagnosis of septic shock and bad treatment outcome. Hypoxia of enterocytes resulting from poor intestinal perfusion in combination with the direct effects of the SARS-CoV-2 virus may lead to increased intestinal permeability and impairment of the intestinal immune barrier function. In patients with septic shock, hypoperfusion of the intestinal mucosa is a potential mechanism of multi-organ failure [63]. In an animal model of shock, Chang et al. showed severe damage to the intestinal barrier, which included the injury and atrophy of the intestinal mucosa and increased intestinal permeability [64]. Shock-associated intestinal ischemia may induce enterocyte damage. Piton et al. showed that almost half of critically ill patients had enterocyte damage on admission to the ICU, as evidenced by elevated levels of I-FABP in the plasma [38]. In our study, 40% of patients were diagnosed with septic shock on ICU admission, and the concentration of I-FABP was much higher in this group of patients compared with patients without shock throughout the study period, while the concentration of citrulline was similar in both groups. This indicates that impaired blood flow in septic shock can lead to the damage of intestinal cells and the release of cytosolic proteins into the bloodstream.

The overall mortality from COVID-19-induced sepsis proved to be higher than from previous viral-induced sepsis [65,66,67,68,69,70]. In our study, the mortality rate was high, and considering the mid-pandemic crisis management, prompted the need for evaluating survival chances of individual patients in the ICU. With 57% mortality in this study vs. 30–60% in patients admitted to the ICU as reported by previous studies, our result is relatively high. It should be noted, however, that during the pandemic there were large differences in the availability of ICU beds, which significantly influenced the admission time and the clinical condition of patients admitted to the ICU, important factors affecting the outcome of treatment. Rapid deterioration of a patient’s health requires the implementation of advanced therapies, often limited to large clinical centers and not accessible to every patient. Therefore, it is important to be able to make decisions early in the first days after ICU admission, and a model that could identify patients with viral sepsis who are at high risk of death would be important in planning effective treatment for these patients. In the search for such a model, intestinal biomarkers are being analyzed based on the fact that gastrointestinal symptoms are one of the major problems in critically ill COVID-19 patients, often complicating therapy by limiting therapeutic options [71].

I-FABP and citrulline levels have been shown to correlate with outcome in critically ill patients [38,40,72]. We found that the I-FABP level measured on admission to the ICU predicted mortality with an AUC of 0.710 and with an optimal cut-off point of 668.57 pg/mL. Moreover, according to the results of multivariate logistic regression analysis, the best model predicting mortality included initial I-FABP, creatinine, and PCT, a diagnosis of septic shock on ICU admission, and the APACHE II score. An initial I-FABP above 668.57 pg/mL, a high APACHE II score, and the presence of septic shock indicated a significantly higher risk of 28-day mortality in the analyzed cohort; the initial PCT and creatinine levels had no statistical significance in the model. In recent studies multiple models have been used to predict mortality in COVID-19 patients.

In a study by Leoni et al. focusing on critically-ill ICU patients, a multivariable mortality prediction model identified age, obesity, procalcitonin, the SOFA score and PaO_2_/FiO_2_ as significant elements associated with 28-day mortality [73]. Our study did not confirm these findings. The observed differences between prediction models were often related to the clinical condition of the studied cohort and the parameters included in the multivariate analysis. All patients in our study required immediate respiratory support in the form of mechanical ventilation or ECMO, while in the Leoni study 27% of patients were treated with less invasive methods of respiratory support (such as CPAP—ventilation using continuous positive airway pressure) on admission to the ICU. It should be emphasized that the new parameter, i.e., the level of I-FABP, included in our model confirms the importance of examining the degree of intestinal wall damage in predicting mortality in COVID-19 patients.

### Limitation

This study had several limitations. The analysis was carried out on the basis of data from one facility and we realize that the criteria for admission to the ICU in our hospital may differ from the criteria of other hospitals. During the pandemic, our ICU acted as the regional ECMO center and only admitted COVID-19 patients with the most severe respiratory symptoms. The results obtained are, therefore, particularly relevant to critical COVID-19 patients who required mechanical ventilation or ECMO.

## 5. Conclusions

In critically ill COVID-19 patients, intestinal cell damage is associated with shock and 28-day mortality. Our findings underline the importance of assessing the degree of intestinal wall damage in predicting patient mortality on ICU admission. Assessing mortality risk based on intestinal markers might be useful in clinical decisions concerning intestinal injury, imaging diagnostics and potential surgical interventions in isolated patients. It should be noted, however, that mortality may depend on the severity of the patients’ condition in the hospital, staff experience, and available resources, which can vary significantly between ICUs.

## Figures and Tables

**Table 1:** Characteristics of the study population.

Parameter	Nonsurvivors, N = 23	Survivors, N = 17	*p*
Age (years)	65 ± 3 (39–90)	53 ± 3 (28–75)	0.025
Sex, male N(%)	16 (69)	9 (53)	0.283
APACHE II score	19 ± 1 (9–33)	14 ± 1 (6–24)	0.018
SOFA score	9 ± 1 (3–13)	7 ± 1 (4–12)	0.126
Comorbidities *n* (%)			
Hypertension	3 (13)	6 (35)	0.100
Coronary heart disease	6 (26)	2 (12)	0.239
Chronic kidney disease	6 (26)	0	0.026
Diabetes	3 (13)	2 (12)	0.645
Obesity	1 (4)	3 (17)	0.197
COPD	1 (4)	0	0.575
BMI	31 ± 2 (20–42)	29 ± 1 (25–37)	0.395
Length of stay, ICU (day)	13 ± 2 (3–44)	27 ± 6 (2–106)	0.049
Length of stay, hospital (day)	16 ± 2 (3–47)	29 ± 4 (10–61)	0.004

APACHE II, Acute Physiology and Chronic Health Evaluation II; SOFA, Sequential Organ Failure Assessment; COPD, Chronic Obstructive Pulmonary Disease; BMI, Body Mass Index; ICU, Intensive Care Unit. Mean ± SE (min-max) and percentage of the total.

**Table 2:** Laboratory findings and treatments at ICU admission.

Parameter	Nonsurvivors, N = 23	Survivors, N = 17	*p*
PCT (ng/mL)	1.05 ± 0.24 (0.03–3.89)	0.43 ± 0.11 (0.05–1.58)	0.156
CRP (mg/L)	159 ± 24 (17–412)	174 ± 23 (11–342)	0.603
WBC (10^3^/uL)	13.2 ± 1.0 (7.1–24.0)	14.6 ± 1.8 (5.7–34.5)	0.681
Platelets (10^3^/uL)	237 ± 21 (60–505)	293 ± 35 (111–553)	0.373
D-dimer (mg/L)	15.27 ± 4.97 (0.54–87.15)	14.33 ± 6.7 (0.47–98.12)	0.198
Fibrinogen (g/L)	5.46 ± 0.46 (2.23–9.04)	6.03 ± 0.5 (2.54–10)	0.493
Creatinine (mg/dL)	1.29 ± 0.13 (0.47–2.66)	0.86 ± 0.10 (0.46–1.87)	0.015
PaO_2_/FiO_2_	150 ± 12 (52–282)	145 ± 14 (61–252)	0.902
Bilirubin (mg/dL)	0.9 ± 0.1 (0.2–3.9)	1.0 ± 0.2 (0.4–2.8)	0.758
Ferritine (ng/mL)	1368 ± 392 (443–3474)	714 ± 237 (71–1725)	0.201
Lactate (mmol/L)	2.21 ± 0.21 (0.70–5.20)	1.51 ± 0.15 (0.70–0.15)	0.024

PCT, Procalcitonin; CRP, C-Reactive Protein; WBC, White Blood Cell; PaO_2_/FiO_2_, arterial oxygen partial pressure (PaO_2_ in mmHg) to fractional inspired oxygen (fraction). Data are presented as Mean ± SE (min-max).

**Table 3:** Incidence of acute gastrointestinal injury based on the AGI score (AGI grade I/II and AGI grade III/IV) and incidence of feeding intolerance assessed on days 1–5.

Parameter	Day 1	Day 2	Day 3	Day 4	Day 5
AGI score (%)					
grade I/II	100	93	90	97	92
grade III/IV	0	7	10	3	8
Feeding intolerance (%)	8	10	10	19	9

**Table 4:** Univariate and multivariate logistic regression analysis of the predictors of 28-day mortality.

Univariate Analysis			Multivariate Analysis	
	Odds Ratio	*p*	Odds Ratio	*p*
APACHE II	1.165730 × 10^0^	0.018	1.583274 × 10^0^	0.018
shock	1.166667 × 10^1^	0.004	8.958130 × 10^1^	0.020
I-FABP	7.428571 × 10^0^	0.005	5.114207 × 10^1^	0.030
age	1.062695 × 10^0^	0.018		
sex	4.921875 × 10^−1^	0.286		
BMI	1.066471 × 10^0^	0.422		
SOFA	1.234788 × 10^0^	0.125		
PCT	2.708447 × 10^0^	0.006	3.078094 × 10^1^	0.187
CRP	9.986929 × 10^−1^	0.669		
WBC	1.002122 × 10^0^	0.971		
Creatinine	5.259518 × 10^0^	0.024	2.216123 × 10^2^	0.685
Citrulline	9.998672 × 10^−1^	0.995		
Lactate	2.901363 × 10^0^	0.057		
PaO_2_/FiO_2_	1.001565 × 10^0^	0.078		

APACHE II, Acute Physiology and Chronic Health Evaluation II; I-FABP, Intestinal Fatty Acid Binding Protein; PCT, Procalcitonin; CRP, C-Reactive Protein; WBC, White Blood Cell; PaO_2_/FiO_2_, arterial oxygen partial pressure (PaO_2_ in mmHg) to fractional inspired oxygen (fraction).

## Data Availability

The data presented in the study are available on request from the corresponding author. The data have not been made publicly available because they contain information that could compromise the privacy of the study participants.
